# Sex and ethnic differences in unrecognized myocardial infarctions: Observations on recognition and preventive therapies from the multiethnic population-based HELIUS cohort^☆^

**DOI:** 10.1016/j.ijcrp.2024.200237

**Published:** 2024-01-12

**Authors:** Bryn Hummel, Julie A.E. van Oortmerssen, CharlotteS.M. Borst, Ralf E. Harskamp, Henrike Galenkamp, Pieter G. Postema, Irene G.M. van Valkengoed, IMPRESS consortium

**Affiliations:** a Department of Public and Occupational Health, Amsterdam UMC, Location AMC, Amsterdam, the Netherlands; b Department of Epidemiology, Erasmus MC, Rotterdam, the Netherlands; c Department of General Practice, Amsterdam UMC, Location AMC, Amsterdam, the Netherlands; d Amsterdam Public Health, Health Behaviours and Chronic Diseases, Amsterdam, the Netherlands; e Departments of Experimental and Clinical Cardiology, Heart Center, Amsterdam UMC Location AMC, Amsterdam, the Netherlands

**Keywords:** Unrecognized myocardial infarction, Tertiary prevention, Sex differences, Ethnic differences, The HELIUS study

## Abstract

**Background:**

Epidemiological studies suggest sex differences in the prevalence and characteristics of unrecognized and recognized myocardial infarction (uMI, rMI). Despite increasingly diverse populations, observations are limited in multiethnic contexts. Gaining better understanding may inform policy makers and healthcare professionals on populations at risk of uMI who could benefit from preventive measures.

**Methods:**

We used baseline data from the multiethnic population-based HELIUS cohort (2011–2015; Amsterdam, the Netherlands). Using logistic regressions, we studied sex differences in the prevalence and proportion of uMIs across ethnic groups. Next, we studied whether symptoms, clinical parameters, and sociocultural factors were associated with uMIs. Finally, we compared secondary preventive therapies in women and men with a uMI or rMI. We relied on pathological Q-waves on a resting electrocardiogram as the electrocardiographic signature for (past) MI.

**Results:**

Overall, and in Turkish and Moroccan subgroups, the prevalence of uMIs was higher in men than women. The proportion of uMIs was similar in women (21.0%) and men (18.4%), yet varied by ethnicity. In women and men, symptoms (chest pain, dyspnea) and clinical parameters (hypertension, hypercholesterolemia), and in women also lower educational level and diabetes were associated with lower odds of uMIs. Women (0.0%) and men (3.6%) with uMI were unlikely to receive secondary preventive therapies compared to those with rMI (28.1–40.9%).

**Conclusions:**

The prevalence of uMIs was higher in men than women, and sex differences in the proportion of uMIs varied somewhat across ethnic groups. People with uMIs did not receive adequate preventative medications, posing a risk for recurrent events.

## 1 Introduction

Myocardial infarctions (MIs) are the most common form of ischemic heart disease, the leading cause of mortality in Europe [1]. An estimated 20–60% of MIs remain clinically undiagnosed, and thus unrecognized, during the acute phase [[2], [3], [4], [5], [6], [7]]. Eventually, these initially unrecognized MIs (uMIs) may be detected, typically, through pathological Q-wave abnormalities indicative of a prior transmural MI [2] on electrocardiography (ECG). These ECG abnormalities, although indicative, warrant further diagnostic work-up to establish the final diagnosis.

As MIs are associated with increased risk of recurrent events and mortality [2], Dutch guidelines recommend medication for the prevention of such cardiovascular complications [[8], [9]]. Yet, studies suggest those with a uMI may be less likely to receive guideline-recommend care than those with a recognized MI (rMI) [10]. As such, individuals with a uMI may be at increased risk for cardiovascular complications [2].

This emphasizes the importance of studying populations at risk of uMIs; yet, contemporary studies are mostly limited to ethnic majority populations, despite evidence suggesting ethnic differences in the prevalence of MIs [11]. The available literature suggests that, while the prevalence of uMIs is higher in men, the proportion of uMIs – among those with an MI - is greater in women [[2], [4], [6], [7], [12]]. However, whether this holds true across different ethnic groups is yet unknown.

The greater proportion of uMIs in women than men may be explained by underrecognition related to sex differences in symptom presentation [13]. Additionally, sociocultural factors, such as educational level, literacy, health literacy, and specifically in ethnic minority groups, cultural distance to the healthcare system, have been associated with healthcare seeking delays in symptomatic patients [14]. We hypothesize these factors may also be associated with the odds of having a uMI [[15], [16]].

Hence, in this study, we will investigate whether sex differences in the prevalence and proportion of uMIs are consistent across ethnic groups. Second, to see which factors may contribute to having uMIs versus rMIs, we will determine whether patient-characteristics, specifically symptoms [17,18], clinical parameters [19], and sociocultural factors [14] are associated with uMIs in women and men in a multiethnic population. Finally, to study to what extent women and men with uMIs miss out on treatment, we will study sex differences in secondary preventive medication use in individuals with uMIs versus rMIs.

## 2 Methods

### 2.1 Sample and population

We conducted a cross-sectional analysis using baseline data from the population-based prospective HELIUS study (Amsterdam, the Netherlands). Participants were randomly recruited from the municipal registry, stratified by ethnicity: Dutch, Surinamese, Ghanaian, Turkish or Moroccan background. Baseline data (2011–2015) were obtained through a questionnaire, physical examination and biological samples. Additionally, participants were requested to bring any prescribed medication to their appointment, to be classified according to Anatomical Therapeutic Chemical (ATC) codes by a trained nurse. A complete description of HELIUS is available elsewhere [20]. All participants provided written informed consent. The HELIUS study was approved by the Institutional Review Board of the Academic Medical Centre.

Of 24,782 HELIUS participants, questionnaire and physical examination data were available for 22,162 participants. We excluded those of Javanese Surinamese origin, other/unknown Surinamese origin, or other/unknown ethnicity (n = 548) due to too low power for subgroup analyses, leaving 21,614 participants to study the prevalence of uMIs and analyses in the total population. Further analyses were conducted in participants with any MI (n = 491). The definitions used are described in brief below and more extensively in Supplemental Table 1.

### 2.2 (Un)recognized MIs

We defined uMIs as major Q-wave abnormalities indicative of a previous transmural MI, detected from standard 12-lead resting ECGs made with a GEMAC5500 type at 500 samples/second and analyzed using ‘Modular ECG Analysis System’. ECG abnormalities were analyzed through Minnesota coding, the GE Marquette 12SL report, and cardiologist assessment [21]. We recognize the limitations of ECGs and self-report data without validating these through other imaging techniques and medical records, and elaborate on this in the discussion.

Participants with either a self-reported MI in the questionnaire, and/or major Q-wave abnormalities on the ECG, were classified as the combined category ‘any MI’. Those with major Q-wave abnormalities, without a self-reported MI, were considered to have had a uMI. Participants with a self-reported MI were considered to have had an rMI, regardless of their ECG findings [22].

### 2.3 Sociodemographic characteristics

Age (in years) and sex (female or male) were derived from the municipality registry.

Ethnicity was defined based on participants' and their parents' registered country of birth [23]. Surinamese participants were further classified according to self-reported ethnic origin into ‘African’, ‘South-Asian’, ‘Javanese’, or ‘other’.

### 2.4 Symptoms

Chest pain was measured via the Rose Angina Questionnaire. We differentiate between any chest pain and severe chest pain [24]. Dyspnea was measured by asking participants if they ever experienced shortness of breath.

### 2.5 Clinical parameters

Diabetes, hypertension and hypercholesterolemia were defined based on self-report, medication, and baseline measurements.

As medication may have been prescribed after a cerebrovascular accident (CVA) [25,26], or for previously diagnosed hypertension or hypercholesterolemia, differences in the prevalence of these factors may have affected differences in secondary preventive therapies between those with a uMI versus rMI. We defined a prior CVA, and awareness of hypertension and hypercholesterolemia based on self-report.

Obesity was defined as a Body Mass Index (BMI) ≥30. BMI was calculated as weight (kg) divided by height squared (m^2^), with weight and height measured in duplicate during physical examinations.

### 2.6 Sociocultural factors

Educational level, defined as the highest obtained educational degree in the Netherlands or country of origin, was used as an indicator of socioeconomic status.

Difficulty with the Dutch language was defined as perceived difficulty in conversations and reading in Dutch.

Health literacy was measured through a Dutch adaptation of Chew's Set of Brief Screening Questions (SBSQ) [[27], [28]].

Cultural distance to the Dutch healthcare system was measured in non-Dutch origin participants based on familiarity with and preference for the healthcare system either in the Netherlands or in their country of origin.

### 2.7 Secondary preventive medication

Adequate secondary preventive medication was defined according to European and Dutch guidelines [8,29] and data availability, as receiving platelet aggregation inhibitors or P2Y12 inhibitors, lipid modifying drugs, and β-blockers. We also described other medications which may be prescribed after an MI: antihypertensive agents, glucose-lowering medication, and vitamin K antagonists, direct factor Xa inhibitors, and/or direct oral anticoagulants Dabigatran.

### 2.8 Statistical analyses

We imputed missing data on all variables through Multiple Imputation via Chained Equations, using five imputations and ten iterations. Variables were used for imputation if no more than 60% of cases was missing, and if the correlation with the variable(s) to be imputed was ≥10%.

Population characteristics are presented as frequencies [%] or medians [interquartile range (IQR)] for women and men in total, and by ethnicity. We calculated the prevalence of any MI and uMIs by sex and ethnicity. Using age- and ethnicity-adjusted logistic regressions, we studied sex differences in the prevalence of uMIs in total, and by ethnicity. Next, to test if sex differences were consistent across ethnic groups, we studied the interaction between sex and ethnicity in uMI prevalence, and compared the model with and without the interaction term via likelihood-ratio tests.

Subsequent analyses were restricted to participants with any MI. First, we described the proportion of uMIs of all MIs by sex and ethnicity. Second, we calculated sex differences in the proportion of uMIs, in total and by ethnicity, using age- and ethnicity-adjusted logistic regressions. We tested if sex differences were consistent by ethnicity, and compared the models with and without the interaction term of ethnicity*sex.

Third, we used age- and ethnicity-adjusted logistic regression analyses, to study if patient-characteristics (chest pain, severe chest pain, dyspnea, diabetes, hypertension, hypercholesterolemia, educational level, difficulty with the Dutch language, health literacy, and cultural distance to the Dutch healthcare system) were associated with having a uMI versus an rMI. These analyses were stratified by sex but not ethnicity, due to too low power for ethnic subgroup analyses.

Fourth, we described secondary preventive therapies in women and men with uMIs or rMIs. Via logistic regression analyses, adjusted for age, ethnicity, a prior CVA, and awareness of hypertension and hypercholesterolemia, we studied sex differences in the association between uMIs and adequate secondary preventive medication. Due to too low power, this analysis was not stratified by ethnicity. Analyses were conducted in RStudio version 4.0.3, with statistical significance defined at p < .05.

### 2.9 Sensitivity analyses

First, using age- and ethnicity-adjusted logistic regressions, we compared how patient-characteristics were associated with uMIs or with rMIs in women and men in the total population.

Second, we explored to what extent our findings were affected by our definition of rMI. First, we defined rMI as a self-reported percutaneous coronary intervention (PCI) or bypass surgery, and uMI as major Q-wave abnormalities in those who did not report a PCI or bypass surgery. Second, in those with available follow-up data (n = 10,362), we defined rMI as a self-reported MI at both baseline and follow-up, hypothesizing this may increase the reliability of self-reported MIs. A uMI, then, was defined as major Q-wave abnormalities in those without a self-reported MI at either baseline or follow-up.

Third, given discussions on the validity of ECG-abnormalities across sexes and ethnicities, we determined the prevalence of uMIs among an ‘apparently healthy’ population. This, as a high prevalence of uMIs in sex- or ethnic subgroups who are otherwise healthy, could suggest major Q-wave abnormalities may not be a valid measure of prior MIs across sexes or ethnic groups. In line with earlier work, we defined ‘apparently healthy’ as no 1) diagnosed arterial disease, 2) diabetes, 3) hypertension, 4) chronic kidney damage, and 5) possible ECG‐modifying medication [30]. Finally, as the validity of ECGs may be compromised in obese populations, we repeated the analyses in a population without obesity.

## 3 Results

Our sample comprised 21,614 participants, of which 12,486 were women (57.8%; Table 1). The median age [IQR] was 45 [33, 54] in women and 46 [34, 55] in men. Of all women, 10.0% had diabetes, 34.6% hypertension, and 24.4% hypercholesterolemia, compared to 12.0%, 41.1%, and 28.7% in men. A prior CVA was reported by 5.4% of women and 4.9% of men, and 31.1% of women and 17.3% of men had obesity. The prevalence of clinical parameters also varied by ethnicity; for instance, 2.4–5.1% of Dutch women and men had diabetes, compared to 17.8–21.6% of South-Asian Surinamese women and men.

**Table 1:** Age, symptoms, and clinical parameters by sex in total, and by ethnicity.

	Total	Dutch	South-Asian Surinamese	African Surinamese	Ghanaian	Turkish	Moroccan
**Women**	12,486	2475	1671	2535	1433	1980	2392
Median age [IQR]	45 [33, 54]	47 [32, 57]	48 [37, 56]	50 [40, 57]	45 [37, 51]	41 [30, 49]	39 [28, 50]
**Symptoms**							
Chest pain	4095 [33.0]	502 [20.3]	671 [40.3]	866 [34.3]	352 [25.0]	776 [39.4]	928 [38.9]
Severe chest pain	1148 [9.3]	71 [2.9]	195 [11.8]	247 [9.8]	114 [8.2]	279 [14.2]	242 [10.2]
Dyspnea	3963 [32.0]	563 [22.8]	660 [39.7]	813 [32.3]	196 [13.9]	856 [43.7]	875 [36.7]
**Clinical parameters**							
Diabetes Mellitus*	1245 [10.0]	59 [2.4]	296 [17.8]	307 [12.2]	138 [9.7]	185 [9.4]	260 [10.9]
Hypertension*	4309 [34.6]	577 [23.4]	674 [40.5]	1284 [50.8]	746 [52.2]	522 [26.4]	506 [21.2]
Hypercholesterolemia*	3040 [24.4]	610 [24.7]	590 [35.4]	664 [26.3]	321 [22.4]	481 [24.3]	375 [15.7]
CVA	670 [5.4]	81 [3.3]	113 [6.8]	170 [6.7]	59 [4.1]	127 [6.4]	120 [5.0]
Obesity	3883 [31.1]	250 [10.1]	391 [23.4]	953 [37.7]	634 [44.3]	813 [41.1]	842 [35.2]
**Men**	9128	2089	1371	1616	905	163	1514
Median age [IQR]	46 [34, 55]	48 [35, 59]	46 [33, 56]	51 [40, 58]	49 [41, 55]	42 [31, 50]	42 [32, 52]
**Symptoms**							
Chest pain	2634 [29.0]	471 [22.6]	508 [37.3]	385 [23.9]	183 [20.5]	580 [35.9]	507 [33.7]
Severe chest pain	710 [7.9]	75 [3.6]	156 [11.5]	104 [6.5]	45 [5.1]	172 [10.7]	158 [10.6]
Dyspnea	2077 [22.9]	392 [18.8]	437 [32.0]	298 [18.5]	78 [8.7]	477 [29.5]	395 [26.3]
**Clinical parameters**							
Diabetes Mellitus*	1089 [12.0]	106 [5.1]	295 [21.6]	187 [11.7]	134 [14.9]	183 [11.3]	184 [12.2]
Hypertension*	3746 [41.1]	776 [37.2]	617 [45.0]	808 [50.2]	563 [62.2]	535 [32.9]	447 [29.6]
Hypercholesterolemia*	2612 [28.7]	596 [28.6]	577 [42.1]	404 [25.0]	268 [29.7]	477 [29.3]	291 [19.3]
CVA	449 [4.9]	79 [3.8]	99 [7.2]	91 [5.6]	36 [4.0]	69 [4.2]	75 [5.0]
Obesity	1582 [17.3]	212 [10.2]	186 [13.6]	278 [17.2]	159 [17.6]	457 [28.0]	290 [19.2]

All data are presented as n [%], unless specified otherwise. Table based on imputed data. IQR, Interquartile Range; CVA, Cerebrovascular Accident. *Based on self-report, baseline measurements, and medication use.

### 3.1 Sex difference in the prevalence of uMIs

The prevalence of uMIs was 0.3% in women and 0.6% in men (Table 2). Women had lower odds of uMIs than men (OR 0.50 [95%CI 0.33, 0.75, p < .001]). Across ethnic groups, uMI prevalence ranged between 0.1% in Ghanaian women to 0.6% in South-Asian Surinamese women, and between 0.4% in African Surinamese men to 0.9% in Turkish men. The uMI prevalence appeared higher in men than women across ethnic groups, with the exception of South-Asian Surinamese, yet, the adjusted odds of uMIs was only statistically significantly higher in Moroccan (0.18 [0.05, 0.62], p = .007) and Turkish (0.29 [0.09, 0.94], p < .039) men than women. The interaction between sex and ethnicity did not improve the model (p = .233), and did not provide evidence that sex differences in the prevalence of uMIs varied by ethnicity.

**Table 2:** Sex- and ethnic differences in the prevalence and proportion of unrecognized myocardial infarctions.

Total	Prevalence					Proportion			
	Any MI of total [%]	uMI [% of total]	Sex difference uMIs within groups		Interaction sex and ethnicity	uMIs of all MIs	Sex difference uMIs within groups		Interaction sex and ethnicity
			OR [95%CI]	P-value	P-value	[%]	OR [95%CI]	P-value	P-value
Women	176 [1.4]	37 [0.3]	0.51 [0.34, 0.76]	.001		21.0	1.08 [0.66, 1.75]	.757	
Men	315 [3.5]	58 [0.6]				18.4			
**Dutch**									
Women	26 [1.1]	11 [0.5]	0.56 [0.26, 1.21]	.139		42.3	1.20 [0.39, 3.62]	.754	
Men	60 [2.9]	17 [0.8]			(Ref.)	28.3			(Ref.)
**South-Asian Surinamese**									
Women	40 [2.5]	10 [0.6]	1.31 [0.47, 3.70]	.606	.190	25.0	4.98 [1.56, 16.0]	.007	.144
Men	92 [6.9]	6 [0.5]				6.5			
**African Surinamese**									
Women	32 [1.3]	7 [0.3]	0.68 [0.24, 1.96]	.474	.779	21.9	1.55 [0.48, 5.10]	.460	.998
Men	46 [2.9]	7 [0.4]				15.2			
**Ghanaian**									
Women	9 [0.6]	2 [0.1]	0.36 [0.06, 2.03]	.248	.792	22.2	0.45 [0.04, 4.60]	.480	.277
Men	12 [1.4]	4 [0.5]				33.3			
**Turkish**									
Women	47 [2.4]	3 [0.2]	0.18 [0.05, 0.62]	.007	.142	6.4	0.32 [0.09, 1.19]	.083	.052
Men	80 [5.0]	14 [0.9]				17.5			
**Moroccan**									
Women	22 [0.9]	4 [0.2]	0.29 [0.09, 0.94]	.039	.373	18.2	0.32 [0.08, 1.28]	.096	.063
Men	25 [1.7]	10 [0.7]				40.0			

Unless specified otherwise, data are presented as n [%]. Table based on imputed data. uMI, unrecognized Myocardial Infarction; MI, Myocardial Infarction; OR, Odds Ratio; CI, Confidence Interval. All analyses were adjusted for age; analyses in the total population were also adjusted for ethnicity. The type III tests to study the model improvement after adding interaction terms between sex and ethnicity was not statistically significant for the analysis on sex differences in uMI prevalence (p = .233), but was significant for the analysis on sex differences in the proportion of uMIs (p = .011).

### 3.2 Sex difference in the proportion of uMIs

UMIs comprised 21.0% (range 6.4–42.3%) of all MIs in women, compared to 18.4% (range 6.5–40.0%) in men (Table 2). The proportion of uMIs of all MIs appeared higher in women than men of Dutch, South-Asian Surinamese and African Surinamese origin, while a reversed pattern appeared in the Ghanaian, Turkish and Moroccan subgroups. Yet, the sex difference in proportion of uMIs was only statistically significant for the South-Asian Surinamese group (OR 4.9 [95%CI1.56, 16.0], p = .007). The interaction term improved the model (p = .011), showing that the pattern of a greater proportion uMIs of all MIs in Dutch women than men, appeared to be reversed for the Moroccan and Turkish groups.

### 3.3 Characteristics associated with uMI vs rMI

Compared to those with uMIs, those with rMIs appeared to have a higher prevalence of symptoms and clinical parameters (Table 3). For instance, 59.5% of women and 63.8% of men with a uMI had hypertension, compared to 81.3% of women and 81.6% of men with an rMI.

**Table 3:** Sociodemographic characteristics, symptoms, clinical parameters, and sociocultural factors in women and men with an unrecognized or recognized myocardial infarction.

	Women		Men	
	uMI	rMI	uMI	rMI
	n = 37	n = 139	n = 58	n = 257
**Sociodemographic characteristics**				
Median age [IQR]	56 [49, 64]	55 [50, 61]	55 [46, 61]	58 [52, 64]
Ethnicity - Dutch	11 [29.7]	15 [10.8]	17 [29.3]	43 [16.7]
South-Asian Surinamese	10 [27.0]	30 [21.6]	6 [10.3]	86 [33.5]
African Surinamese	7 [18.9]	25 [18.0]	7 [12.1]	39 [15.2]
Ghanaian	2 [5.4]	7 [5.0]	4 [6.9]	8 [3.1]
Turkish	3 [8.1]	44 [31.7]	14 [24.1]	66 [25.7]
Moroccan	4 [10.8]	18 [12.9]	10 [17.2]	15 [5.8]
**Symptoms**				
Any chest pain	6 [16.2]	92 [66.2]	12 [21.1]	146 [56.8]
Severe chest pain	1 [2.7]	52 [37.4]	4 [7.0]	84 [32.7]
Dyspnea	9 [24.3]	84 [60.4]	12 [21.1]	120 [46.7]
**Clinical parameters**				
Diabetes Mellitus	6 [16.2]	60 [43.8]	15 [25.9]	78 [30.4]
Prior CVA	2 [5.4]	26 [19.0]	8 [13.8]	35 [13.6]
Awareness of hypertension*	22 [59.5]	116 [84.7]	37 [63.7]	212 [82.5]
Awareness of hypercholesterolemia*	20 [54.1]	109 [79.6]	26 [44.9]	206 [82.5]
Obesity	14 [37.8]	68 [49.6]	26 [44.8]	52 [20.2]
**Sociocultural factors**				
Low educational level	15 [41.7]	107 [78.1]	34 [58.6]	160 [62.3]
Difficulty with the Dutch language	11 [29.7]	72 [52.6]	20 [34.5]	93 [36.2]
Low health literacy	4 [10.8]	50 [36.5]	7 [12.1]	58 [22.6]
Cultural distance Dutch healthcare system	6 [16.2]	20 [14.6]	4 [6.9]	31 [12.1]

Data are presented as n [%], unless specified otherwise. Table based on imputed data. uMI, unrecognized Myocardial Infarction; rMI, recognized Myocardial Infarction; IQR, Interquartile Range; CVA, Cerebrovascular Accident. *Based on self-report.

In those with any MI, (severe) chest pain, dyspnea, hypertension, and hypercholesterolemia, were similarly associated with lower odds of uMIs in women and men (Table 4). Additionally, in women but not men, diabetes and a lower educational level were associated with lower odds of uMIs.

**Table 4:** Associations between symptoms, clinical parameters, and sociocultural factors with having an unrecognized versus recognized myocardial infarction, in women and men with any myocardial infarction.

	Women		Men	
	OR [95%CI]	P-value	OR [95%CI]	P-value
Chest pain	0.09 [0.03, 0.25]	<.001	0.15 [0.07, 0.32]	<.001
Severe chest pain	0.04 [0.01, 0.38]	.005	0.11 [0.04, 0.34]	<.001
Dyspnea	0.24 [0.10, 0.57]	.001	0.30 [0.14, 0.61]	.001
Diabetes Mellitus*	0.22 [0.08, 0.61]	.004	1.07 [0.53, 2.18]	.841
Hypertension*	0.24 [0.09, 0.64]	.004	0.44 [0.22, 0.89]	.023
Hypercholesterolemia*	0.31 [0.13, 0.75]	.009	0.24 [0.12, 0.47]	<.001
Lower educational level	0.25 [0.10, 0.60]	.002	1.01 [0.51, 2.03]	.973
Difficulty with the Dutch language	0.80 [0.27, 2.35]	.681	0.65 [0.27, 1.59]	.344
Low health literacy	0.34 [0.10, 1.18]	.090	0.46 [0.17, 1.20]	.112
Cultural distance Dutch healthcare system	1.31 [0.42, 4.06]	.635	0.63 [0.19, 2.03]	.435

### 3.4 Secondary preventive medication

β-blockers, lipid modifying drugs and platelet aggregation/p2Y12 inhibitors use was consistently higher in men (ranging between 54.1 and 66.9%) and women (47.5–58.3%) with an rMI, than men (9.2–26.0%) and women (7.9–27.3%) with a uMI (Table 5, Supplemental Table 2). No women, and a small proportion of men (3.6%) with a uMI received adequate secondary preventive medication, compared to 28.1% of women and 40.9% of men with an rMI. In line, men with a uMI had significantly lower adjusted odds of receiving adequate secondary preventive medication than men with an rMI. Because no women with a uMI in our sample received adequate secondary preventive medication, the odds ratio could not be adequately estimated, hence, no formal interaction test was conducted.

**Table 5:** Secondary preventive medication use, in women and men with an unrecognized or recognized myocardial infarction.

	Women				Men			
	uMI	rMI	OR [95%CI]	P-value	uMI	rMI	OR [95%CI]	P-value
Adequate secondary preventive medication	0 [0.0]	39 [28.1]	0.00 [0.00, INF]	.987	2 [3.6]	105 [40.9]	0.05 [0.01, 0.21]	<.001
Β-blockers (C07)	5 [14.1]	66 [48.2]			6 [10.6]	139 [54.1]		
Lipid modifying drugs (C10)	10 [27.3]	81 [59.1]			16 [26.0]	172 [66.9]		
Platelet aggregation/P2Y12 inhibitors (B01AC)	3 [7.9]	71 [51.8]			6 [9.2]	158 [61.5]		

Data are presented as n [%], unless specified otherwise. Table based on imputed data. uMI, unrecognized Myocardial Infarction; rMI, recognized Myocardial Infarction; OR, Odds Ratio; CI, Confidence Interval; CVA, Cerebrovascular Accident. Analyses are adjusted for age, ethnicity, and awareness of hypertension, hypercholesterolemia, and a prior CVA.

### 3.5 Sensitivity analyses

In the total population, symptoms and clinical parameters were associated with higher odds of rMIs, and associations were weaker (clinical parameters) or reversed (symptoms) for uMIs (Supplemental Table 3). While not statistically significant, low health literacy appeared negatively associated with uMIs. The association of low health literacy with rMIs was only statistically significant in men (p = .006), but not in women (p = .077). The sensitivity analyses using alternative definitions of rMIs, and excluding individuals with obesity, did not change our interpretation of these results (Supplemental Table 4). Moreover, consistently across sexes and ethnicities, we found a low prevalence of major Q-wave abnormalities in the ‘apparently healthy’ population (Supplemental Table 5).

## 4 Discussion

The absolute uMI prevalence was higher in men than women, while the relative proportions uMIs of any MI was similar between women and men. These sex differences, however, varied somewhat by ethnicity. We observed that symptoms and clinical parameters, and in women also a lower educational level and diabetes, were associated with a lower odds of uMIs. Both women and men with uMIs were unlikely to receive secondary preventive medication.

One limitation pertains to our use of electrocardiographic criteria for MI to define uMIs. First, not all MIs result in Q-wave abnormalities. Second, studies have shown that ECGs, compared to e.g. cardiac magnetic resonance imaging, have a high specificity, yet low sensitivity to identify MIs [2,21]. Unfortunately, imaging techniques to validate ECG findings were unavailable. Thus, we may have not only missed individuals who experienced smaller Q-wave or non-Q-wave infarctions, but also individuals whose Q-wave abnormalities were not detected on the ECG. Additionally, concerns have been raised about sex- and ethnic disparities in the validity of ECG abnormalities for classifying cardiac pathology, e.g. due to challenges in ECG lead placements in women due to breast tissue [31], and different normal values in ST-segment elevation in people of Sub-Saharan African descent [30,32]. While we found no evidence for sex- and ethnic differences in the validity of Q-wave abnormalities, in literature nor in our analysis in the ‘apparently healthy’ population, available evidence is limited [33,34]. Hence, we recommend future research study the validity of Q-wave abnormalities for identifying uMIs across ethnicities.

A second limitation pertains to our use of self-report data. We could not verify participants’ self-reported MIs or medication use from health records. Thus, our classification of rMIs and medication use may be subject to bias; i.e. participants may have falsely reported (not) having had an MI or receiving medication. Nevertheless, our sensitivity analyses using alternative definitions of rMI yielded similar results to our main analysis, which strengthens our findings.

Similar to previous work, we found a higher prevalence of uMIs in men than women in the total population [[2], [4], [6], [7]], which likely reflects the generally higher prevalence of any MI in men. The higher prevalence of uMIs in men was observed across most ethnic groups, except for the South-Asian Surinamese. This may be explained by greater awareness of the increased risk of cardiovascular disease (CVD) and risk factors among South-Asians [25], particularly South-Asian men, as illustrated by the relatively low proportion of uMIs in South-Asian Surinamese men compared to, e.g., Dutch men.

These differences in awareness of CVD risk in different sex- and ethnic groups may also have been reflected in differences in the proportion of uMIs. While most studies report a greater proportion of uMIs of any MI in women than men, our study shows more mixed results. We found a greater proportion of uMIs among women than men in the Dutch and both Surinamese groups, while the reverse appears true in the Ghanaian, Turkish and Moroccan group. The low symptom prevalence in participants with a uMI, and the different pattern of sex differences in native Dutch speaking (Dutch and Surinamese) versus non-native Dutch speaking (Ghanaian, Turkish, and Moroccan) groups, lead us to believe an explanation may be found in the recognition, interpretation, and communication of symptoms. We recommend future work study the patient- and healthcare system-related factors contributing to MIs going unrecognized, for instance the barriers to care that women and men of different ethnic groups experience [35].

Differences in CVD risk awareness may also explain the associations between diabetes and a lower educational level and uMIs in women. We found diabetes and a lower educational level were associated with *lower* odds of uMIs in women, despite previous work showing those with diabetes were more likely to experience silent ischemia, which is associated with uMIs, as well as research stating that lower socioeconomic status (for which education is often used as an indicator) is associated with decreased healthcare access [36]. The association between diabetes and lower odds of uMIs in our study may be explained by awareness of the higher CVD risk among patients and healthcare professionals and more frequent diabetes-related healthcare checkups. Sex differences in diabetes awareness may explain why we specifically found this association in women: a previous HELIUS publication indeed suggests greater diabetes awareness in women (77.9–89.5%) than men (60.0–83.3%) [37]. For educational level, we speculate higher educated women may have a lower perceived risk of CVD and fewer risk factors, possibly resulting in them less often seeking or receiving care during an MI. Unfortunately, we do not have the data to explore whether this explains these findings. Strategies for identifying those at risk of uMIs should be studied in future work, as this could have clinical implications for prevention and risk management.

In line with earlier work, we found symptoms to be associated with lower odds of uMIs [6,12], indicating symptomatic people are more likely to seek and receive care. While the low symptom prevalence in those with uMIs may be expected, we also found a low symptoms prevalence among those with an rMI compared to earlier studies [17,38]. While 68.3% of women and 60.5% of men with an rMI in our study reported chest pain, this was 92.9%–93.3% of women and men in other studies [17]. This may be explained by the study sample, as, e.g. Ferry et al. [17] interviewed emergency department patients, who may have greater (perceived) symptom severity [39]. Alternatively, recall bias may have affected retrospective symptom reporting in our study, as HELIUS participants were asked about symptoms during baseline measurements, not during or shortly after the MI [40]. Conversely, those with an rMI in our study more often reported dyspnea (49.0–63.6%) compared to earlier work (15.2–23.3%) [17], which may be explained by our measurement of dyspnea ever, versus dyspnea during an MI.

Finally, we studied whether those with uMIs may be at risk of missing out on treatment to prevent cardiovascular complications. Compared to earlier work (46.2% [10]), we found much lower secondary preventive medication use in women and men with a uMI. While we are not certain whether there may have been contraindications to not prescribe medication, these results suggest those with uMIs may not receive adequate secondary preventive treatment. Notably, compared to previous work (79.2% [10]), secondary preventive medication use was also lower for those with an rMI in our study. This proportion may have been underestimated if participants did not bring (all) medication to location, for instance if they do not use medication as prescribed [41]. Alternatively, the low medication use may be explained by our relatively young [42] and multiethnic [43] population, who may not adhere to medication prescriptions due to lower perceived disease severity [42] or cultural barriers to medication (e.g. misbeliefs about medication, miscommunication with healthcare providers [43]). For healthcare professionals, this implies a need to improve secondary preventive medication adherence in patients with MIs, particularly in younger patients or patients with a migration background.

Concluding, similar to the higher prevalence of MIs in men, we found a higher prevalence of uMIs in men than women. However, while the proportion of uMIs of all MI were similar in women and men overall, patterns varied across ethnic groups. In women and men, in particular the absence of symptoms and clinical parameters, but not sociocultural factors, were associated with the odds of MIs having gone unrecognized. Finally, in our multiethnic population, both women and men with uMIs are at increased risk of cardiovascular complications, due to low secondary preventive medication use.

## Data availability

The HELIUS data are owned by the Amsterdam University Medical Centers, location AMC in Amsterdam, The Netherlands. Any researcher can request the data by submitting a proposal to the HELIUS Executive Board as outlined at http://www.heliusstudy.nl/en/researchers/collaboration, by email: heliuscoordinator@amsterdamumc.nl. The HELIUS Executive Board will check proposals for compatibility with the general objectives, ethical approvals and informed consent forms of the HELIUS study. There are no other restrictions to obtaining the data and all data requests will be processed in the same manner.

## CRediT authorship contribution statement

**Bryn Hummel:** Conceptualization, Formal analysis, Writing – original draft, Writing – review & editing. **Julie A.E. van Oortmerssen:** Writing – review & editing. **CharlotteS.M. Borst:** Writing – original draft, Writing – review & editing. **Ralf E. Harskamp:** Conceptualization, Writing – review & editing. **Henrike Galenkamp:** Writing – review & editing. **Pieter G. Postema:** Writing – review & editing. **Irene G.M. van Valkengoed:** Conceptualization, Writing – original draft, Writing – review & editing.

## Declaration of competing interest

The authors report no relationships that could be construed as a conflict of interest.

Table based on imputed data. OR, Odds Ratio; CI, Confidence Interval. All analyses were adjusted for age and ethnicity. *Based on self-report, baseline measurements, and medication use.
