# Supplementary material for: Sex and ethnic differences in unrecognized myocardial infarctions: Observations on recognition and preventive therapies from the multiethnic population-based HELIUS cohort
Source: Int J Cardiol Cardiovasc Risk Prev. 2024 Jan 12;20:200237. doi: 10.1016/j.ijcrp.2024.200237 (PMC10818071; doi:10.1016/j.ijcrp.2024.200237)
Supplement: Multimedia component 1 [file mmc1.docx]

**Supplemental table 1. Definitions of variables in the study**

| *Any myocardial infarction (MI)* | Participants with either a self-reported MI, and/or major Q-wave abnormalities on the electrocardiogram (ECG), were classified as the combined category ‘any MI’. |
| --- | --- |
| *Unrecognized MIs (uMI)* | We defined uMI as major Q-wave abnormalities indicative of a previous transmural MI in people without a self-reported MI. |
| *Recognized MIs (rMI)* | Participants with a self-reported MI were considered to have had a rMI, regardless of their ECG findings^22^. |
| *Major Q-wave abnormalities* | Q-wave abnormalities indicative of a previous transmural MI were detected from standard 12-lead resting ECGs made with a GEMAC5500 type at 500 samples/second and analyzed using ‘Modular ECG Analysis System’. ECG abnormalities were analyzed through Minnesota coding, the GE Marquette 12SL report, and cardiologist assessment. Recommendations of international expert groups determined the final classification in case of discrepancies among these three methods. Major Q-wave abnormalities were determined based on any of the following criteria: 1) leads V2–V3>0.02 s or QS complex in leads V2–V3, 2) Q-wave≥0.03 s and ≥1 mm deep or QS complex in leads I, II, aVL, aVF or V4–V6 in any two leads of a contiguous lead grouping (I, aVL; V1–V6; II, III, aVF), and/or 3) R wave>0.04 s in V1–V2 and R/S>1 with a concordant positive T wave in absence of conduction defect^21^. |
| *Self-reported MI* | Self-reported MIs were measured via the question ‘Have you ever had a heart attack (myocardial infarction)?’. |
| *Age* | Age (in years) at the time of the data collection was determined from the date of birth derived from the municipality registry. |
| *Sex* | Sex (female or male) was derived from the municipality registry. |
| *Ethnicity* | Ethnicity was defined based on participants’ and their parents’ country of birth^23^. Participants were considered to belong to one of the included ethnic minority groups if they, and at least one parent, were born abroad, or if they were born in the Netherlands, but both their parents were born abroad. Surinamese participants were further classified according to self-reported ethnic origin into ‘African’, ‘South-Asian’, ‘Javanese’, or ‘other’. Participants were considered to be of Dutch origin if they, and both their parents, were born in the Netherlands. |
| *Chest pain* | Chest pain was measured via the Rose Angina Questionnaire, and was defined as any self-reported chest pain, excluding symptoms caused by a cold, asthma, or heartburn. |
| *Severe chest pain* | Severe chest pain was defined as chest pain lasting longer than 30 minutes, which may be indicative of a possible MI^24^. |
| *Dyspnea* | Dyspnea was measured by asking participants if they ever experienced shortness of breath, excluding dyspnea caused by a cold. |
| *Diabetes* | Diabetes was defined based on self-report, medication, and baseline measurements. In addition to self-report, we considered fasting plasma glucose ≥7mmol/L^44^ and/or medication classes A10 for diabetes. Fasting blood samples were drawn after an overnight fast to determine fasting plasma glucose. Medication was registered at the time of the HELIUS visit; to which participants were asked to bring all medication. |
| *Hypertension* | Hypertension was defined based on self-report, medication, and baseline measurements. Awareness of hypertension was classified based on self-report. In addition to self-report, we considered systolic blood pressure ≥140mmHg, diastolic blood pressure ≥90mmHg, and/or medication classes C02, C03, C07, C08, and C09 for hypertension. Systolic and diastolic blood pressure were measured in duplicate on participants’ left arm using an automated digital BP device after participants had been sitting for five minutes. Medication was registered at the time of the HELIUS visit; to which participants were asked to bring all medication. |
| *Hypercholesterolemia* | Hypercholesterolemia was defined based on self-report, medication, and baseline measurements. Awareness of hypercholesterolemia was defined based on self-report. In addition to self-report, we considered total cholesterol ≥6.2mmol/L and/or medication classes C10 for hypercholesterolemia. Fasting blood samples were drawn after an overnight fast to determine. Medication was registered at the time of the HELIUS visit; to which participants were asked to bring all medication. |
| *Prior cerebrovascular accident (CVA)* | We defined a prior CVA based on self-reported CVA, which was asked via the question: “have you ever had a stroke (a cerebral infarction or a cerebral hemorrhage)?”. |
| *Awareness of hypertension* | Awareness of hypertension was defined as self-reported diagnosed hypertension, asked via the question: “has a medical professional ever diagnosed you with high blood pressure?”. |
| *Awareness of hypercholesterolemia* | Awareness of hypercholesterolemia was defined as self-reported diagnosed hypercholesterolemia, asked via the question: “has a medical professional ever diagnosed you with high blood cholesterol?”. |
| *Obesity* | Obesity was defined as a Body Mass Index ≥30. Body Mass Index was calculated as weight (kg) divided by height squared (m^2^), with weight and height measured in duplicate, with the participant in light clothing, during physical examinations. |
| *Education level* | Educational level, defined as the highest obtained educational degree in the Netherlands or country of origin, was used as an indicator of socioeconomic status. Educational level was categorized into lower (no or elementary education, lower vocational, or lower secondary education), and intermediate/higher education (intermediate vocational or secondary education, higher vocational education, or university). |
| *Difficulty with the Dutch language* | Difficulty with the Dutch language was defined as perceived difficulty in conversations and reading in Dutch. This was measured through two questions, e.g.: “Do you experience difficulty in understanding the language when you read the newspaper, a letter, or a leaflet in Dutch?”. Difficulty with the Dutch language was defined as participants answering yes to one or both questions. |
| *Health literacy* | Health literacy was measured through a Dutch adaptation of Chew’s Set of Brief Screening Questions (SBSQ)^27^. The SBSQ consists of three statements on participants’ health literacy, e.g.: “How confident are you filling out medical forms by yourself?”, to be answered on five-point Likert scales. According to Chew's cut-off point, a mean score <3 indicates low health literacy^28^. |
| *Cultural distance to the Dutch healthcare system* | Cultural distance to the Dutch healthcare system was measured in non-Dutch origin participants familiar with the healthcare system in their country of origin. They were asked five two-fold questions concerning differences between the healthcare system in the Netherlands versus in their country of origin, e.g.: “Do doctors in (country of origin) treat patients differently than doctors in the Netherlands?”. Answers included “Yes, very (score 2)/a little differently (1)”, “No, no difference (0)”, or “I do not know” (missing). Participants who answered “yes, very/a little differently” were asked which they prefer, answered as: “the (country of origin) way is much a little (2)/much (3) more pleasant”, “no preference” (0), “the Dutch way is a little (-2)/much (-3) more pleasant”. Scores on the two-fold questions were multiplied, resulting in scores ranging between -6 to +6, which were then averaged for the five statements. Scores <0 were defined as cultural distance to the Dutch healthcare system. Scores were set to 0 for Dutch origin participants and participants without experience with the healthcare |
| *Adequate secondary preventive medication* | The European Society of Cardiology guideline^29^ and the Dutch General Practitioner Society guideline for acute coronary syndrome^8^ recommend prescribing P2Y12-inhibitors, lipid modifying drugs, β-blockers, acetylsalicylic acid, and ACE-inhibitors, following a MI. Based on these guidelines and data availability, we defined adequate secondary preventive medication as a participant receiving platelet aggregation inhibitors or P2Y12 inhibitors (B01AC), lipid modifying drugs (C10), and β-blockers (C07). |
| *Other secondary preventive medication* | We also described other medications which may be prescribed after a MI: antihypertensive agents consisting of diuretics (C03), calcium channel blockers (C08), renin–angiotensin system (C09) and other antihypertensive agents (C02), glucose-lowering medication (A10), and vitamin K antagonists, direct factor Xa inhibitors, and/or direct oral anticoagulants Dabigatran (B01AA/B01AF/ B01AE07). |

**Supplemental table 2. Medication use of other medication that may be prescribed after a myocardial infarction, by sex and unrecognized or recognized myocardial infarction.**

|  | Women | | Men | |
| --- | --- | --- | --- | --- |
|  | uMI | rMI | uMI | rMI |
|  | n=37 | n=137 | n=58 | n=257 |
| Other antihypertensive medication |  |  |  |  |
| Antihypertensive medication (C02) | 1 [2.6] | 3 [2.2] | 0 [0.0] | 3 [1.2] |
| Diuretics (C03) | 2 [5.7] | 29 [21.2] | 1 [2.0] | 29 [11.3] |
| Calcium channel Blockers (C08) | 8 [21.6] | 26 [19.0] | 4 [6.7] | 58 [22.6] |
| RAAS-inhibitors (C09) | 11 [30.0] | 65 [47.4] | 21 [34.4] | 126 [49.0] |
| Glucose-lowering medication (A10) | 2 [5.3] | 51 [37.2] | 11 [19.0] | 63 [24.5] |
| Vitamin K antagonists, direct factor Xa inhibitors, and/or direct oral anticoagulants Dabigatran (B01AA/ B01AF/B01AE07) | 0 [0.0] | 6 [4.4] | 0 [0.3] | 17 [6.6] |

Data are presented as n [%]. Table based on imputed data. uMI, unrecognized Myocardial Infarction; rMI, recognized Myocardial Infarction; RAAS, Renin Angiotensin Aldosterone System.

**Supplemental Table 3. Symptoms, clinical parameters and sociocultural factors associated with unrecognized or recognized myocardial infarctions versus no myocardial infarction, in women and men in the total population.**

|  | Women | | | | Men | | | |
| --- | --- | --- | --- | --- | --- | --- | --- | --- |
|  | uMI | | rMI | | uMI | | rMI | |
|  | OR [95%CI] | P-value | OR [95%CI] | P-value | OR [95%CI] | P-value | OR [95%CI] | P-value |
| Chest pain | 0.30 [0.16, 0.96] | .041 | 4.15 [2.87, 6.00] | <.001 | 0.69 [0.36, 1.31] | .260 | 3.81 [2.91, 4.99] | <.001 |
| Severe chest pain | 0.27 [0.04, 2.01] | .204 | 5.72 [3.99, 8.18] | <.001 | 0.95 [0.34, 2.67] | .925 | 6.61 [4.92, 8.88] | <.001 |
| Dyspnea | 0.60 [0.28, 1.27] | .180 | 2.78 [1.95, 3.98] | <.001 | 0.82 [0.43, 1.55] | .541 | 2.65 [2.04, 3.44] | <.001 |
| Diabetes Mellitus* | 0.96 [0.38, 2.42] | .927 | 3.67 [2.52, 5.33] | <.001 | 1.82 [0.97, 3.44] | .063 | 1.53 [1.14, 2.06] | .005 |
| Hypertension* | 1.68 [0.79, 3.56] | .178 | 5.24 [3.25, 8.44] | <.001 | 1.92 [1.08, 3.42] | .026 | 3.68 [2.62, 5.17] | <.001 |
| Hypercholesterolemia* | 1.90 [0.93, 3.90] | .080 | 6.02 [3.90, 9.29] | <.001 | 1.41 [0.81, 2.46] | .230 | 5.18 [3.72, 7.21] | <.001 |
| Lower educational level | 0.63 [0.30, 1.32] | .223 | 2.66 [1.68, 4.21] | <.001 | 1.57 [0.88, 2.79] | .128 | 1.47 [1.10, 1.98] | .010 |
| Difficulty with the Dutch language | 0.93 [0.36, 2.40] | .885 | 1.32 [0.83, 2.10] | .234 | 0.77 [0.36, 1.61] | .484 | 1.02 [0.72, 1.45] | .914 |
| Low health literacy | 0.47 [0.14, 1.53] | .209 | 1.50 [0.96, 2.35] | .077 | 0.63 [0.26, 1.49] | .289 | 1.67 [1.16, 2.39] | .006 |
| Cultural distance Dutch healthcare system | 1.73 [0.68, 4.39] | .249 | 1.30 [0.79, 2.14] | .298 | 0.76 [0.27, 2.17] | .613 | 1.35 [0.89, 2.07] | .161 |

Table based on imputed data. uMI, unrecognized Myocardial Infarction; rMI, recognized Myocardial Infarction; OR, Odds Ratio; CI, Confidence Interval. All analyses are adjusted for age and ethnicity. *Based on self-report, baseline measurements, and medication use

**Supplemental Table 4. Sex differences in the prevalence and proportion of unrecognized infarctions, using a self-reported myocardial infarction at baseline and follow-up, and using a self-reported percutaneous coronary intervention/bypass surgery as alternative definitions of recognized myocardial infarction, and in a population without obesity.**

|  | Prevalence | | | | Proportion | | |
| --- | --- | --- | --- | --- | --- | --- | --- |
|  | uMI | rMI | Sex difference | | uMIs (%) | Sex difference | |
|  |  |  | OR [95%CI] | P-value |  | OR [95%CI] | P-value |
| rMI based on PCI/bypass surgery | | |  |  |  |  |  |
| Total n | 21614 | |  |  |  |  |  |
| Women | 39 [0.3] | 260 [2.1] | 0.51 [0.34, 0.77] | .001 | 13.0 | 1.00 [0.64, 1.56] | .986 |
| Men | 62 [0.7] | 387 [4.3] |  |  | 13.8 |  |  |
|  |  |  |  |  |  |  |  |
| rMI based on baseline + follow up data | | | |  |  |  |  |
| Total n | 10362 | |  |  |  |  |  |
| Women | 14 [0.2] | 22 [0.4] | 0.43 [0.22, 0.82] | .011 | 38.9 | 0.97 [0.44, 2.12] | .939 |
| Men | 26 [0.6] | 83 [1.8] |  |  | 23.9 |  |  |
|  |  |  |  |  |  |  |  |
| Population without obesity | | |  |  |  |  |  |
| Total n | 16149 | |  |  |  |  |  |
| Women | 23 [0.3] | 66 [0.8] | 0.70 [0.41, 1.20] | .193 | 26.0 | 1.78 [0.93, 3.43] | .083 |
| Men | 32 [0.4] | 200 [2.7] |  |  | 14.0 |  |  |

Data are presented as n [%], unless specified otherwise. Table based on imputed data. uMI, unrecognized Myocardial Infarction; rMI, recognized Myocardial Infarction; PCI, Percutaneous Coronary Intervention; OR, Odds Ratio; CI, Confidence Interval. Analyses were adjusted for age and ethnicity.

**Supplemental table 5. Sex differences in the prevalence of unrecognized myocardial infarctions in the ‘apparently healthy’ subset of the population.**

|  | Total | Women | Men | Sex difference prevalence uMI* | | | | |
| --- | --- | --- | --- | --- | --- | --- | --- | --- |
|  |  |  |  | OR [95%CI] | P-value | |  |  |
| Total (n) | 10475 | 6197 | 4278 |  | |  | |  |
| uMI [%] | 29 [0.3] | 13 [0.2] | 16 [0.4] | 0.60 [0.28, 1.29] | | .192 | |  |
| Dutch (n) | 2751 | 1603 | 1148 |  | |  | |  |
| uMI [%] | 9 [0.3] | 4 [0.2] | 5 [0.4] |  | |  | |  |
| South-Asian Surinamese (n) | 1296 | 737 | 559 |  | |  | |  |
| uMI [%] | 5 [0.4] | 4 [0.5] | 1 [0.2] |  | |  | |  |
| African Surinamese (n) | 1626 | 987 | 639 |  | |  | |  |
| uMI [%] | 2 [0.1] | 2 [0.2] | 0 [0.0] |  | |  | |  |
| Ghanaian (n) | 816 | 533 | 283 |  | |  | |  |
| uMI [%] | 1 [0.1] | 0 [0.0] | 1 [0.4] |  | |  | |  |
| Turkish (n) | 1829 | 995 | 834 |  | |  | |  |
| uMI [%] | 4 [0.2] | 1 [0.1] | 3 [0.4] |  | |  | |  |
| Moroccan (n) | 2157 | 1342 | 815 |  | |  | |  |
| uMI [%] | 7 [0.3] | 2 [0.1] | 5 [0.6] |  | |  | |  |

Table based on imputed data. uMI, unrecognized Myocardial Infarction; OR, Odds Ratio; CI, Confidence Interval. Analyses were adjusted for age and ethnicity. *Due to the low prevalence of uMIs in the ‘apparently healthy’ population, we did not calculate sex differences in the prevalence of uMIs by ethnic group.
